# Depression and Cognition Mediate the Effect of Self-Perceptions of Aging Over Frailty Among Older Adults Living in the Community in China

**DOI:** 10.3389/fpsyg.2022.830667

**Published:** 2022-06-16

**Authors:** Kun Yuan, Yanyan Luo, Junjun Sun, Hongjuan Chang, Huijie Hu, Bingwei Zhao

**Affiliations:** ^1^School of Nursing, Xinxiang Medical University, Xinxiang, China; ^2^Faculty of Nursing, Sanquan College of Xinxiang Medical University, Xinxiang, China; ^3^School of Nursing, Huzhou University, Huzhou, China

**Keywords:** self-perceptions of aging, depressive symptoms, cognitive status, frailty, older adults

## Abstract

**Objectives:**

The aims of the study were first to investigate the association between self-perceptions of aging and frailty and second to determine whether self-perceptions of aging affects frailty *via* depressive symptoms and cognitive status among older adults living in the community in China.

**Methods:**

Among 850 older adults who participated in this cross-sectional study, 822 older adults made valid responses to Tilburg Frailty Indicator, Brief Aging Perceptions Questionnaire, UCLA loneliness scale-8, Mini-Mental State Examination, and Patient Health Questionnaire-9 between March to December 2019. The possible pathways of self-perceptions of aging affecting frailty were analyzed based on the structural equation modeling analysis.

**Results:**

A total of 21.53% of older adults reported frailty. Correlation analyses showed that higher degrees of frailty were related to greater loneliness, more depressive symptoms, more negative self-perceptions of aging, worse locomotive function, and cognitive status (*r* = 0.267, *r* = 0.440, *r* = 0.481, *r* = 0.451, *r* = −0.337; *p* < 0.001). Multiple regression analysis showed that loneliness, depressive symptoms, self-perceptions of aging, locomotive function, and cognitive status were the five factors to be entered the regression equation, and the variance of joint explanation was 46.60%. SPA had a direct effect on frailty (*β* = 0.306 and *p* < 0.001), and SPA indirectly affects frailty by independently affecting depressive symptoms (*β* = 0.391, 95% CI [0.027, 0.061], and *p* < 0.001) or cognitive status (*β* = 0.148, 95% CI [0.009, 0.024], and *p* < 0.001) of older adults.

**Conclusion:**

These findings help explain the potential psychological mechanisms through which SPA impacts frailty and may aid community healthcare providers in China in identifying individuals at high risk of frailty. The results suggest that health staff should help older adults improve their perspectives on aging, alleviate or prevent depressive symptoms, and improve cognitive status to delay the progress of frailty and promote healthy aging.

## Introduction

An aging population is an inevitable part of social development. However, the current acceleration of population aging is having a far-reaching impact on the planning and provision of health and social care worldwide (Wu et al., 2017). China has the world’s largest population of adults over 60 years old. The growing number of adults entering old age demonstrates the importance of understanding the processes in and around older adults to ensure that older adults are able to participate actively and not become frailty and/or ill. Frailty is recognized as a clinical syndrome which increased vulnerability and decreased ability to cope with stress caused by the decline of physiological reserves or multiple functional abnormalities that arise in older people, manifested in multiple aspects physically, psychologically, and socially (Walston et al., 2006; Puts et al., 2017). The incidence of frailty among China’s older population is relatively high, and the phenomenon tends to increase with age (Ye et al., 2020). Frailty is an important risk factor leading to various adverse health consequences for older adults, including an increase in the incidence of falls and disability (Kojima, 2015; Makizako et al., 2015). It can also affect their health status, functional integrity, and quality of life-all while bringing a heavy burden to families and society (Arjunan et al., 2018). Since frailty is composed of a constellation of symptoms, it is likely that no single biomarker can perfectly predict this syndrome. Instead, frailty exists in the complex interaction of various factors. In addition, according to the frailty dynamic equilibrium hypothesis, frailty is a potentially reversible process, which can change in either direction with the passage of time (Junius-Walker et al., 2018). Therefore, given the high incidence and prevalence of frailty and the negative health conditions and associated factors, modifiable factors and underlying mechanisms associated with frailty progression need to be explored to reverse the course of frailty in older adults.

According to stereotype embodiment theory (SET), as people age, internalized age-related stereotypes, whether positive or negative, are eventually applied to the ego and translated into expectations and attitudes about a person’s aging process (Levy, 2009). This introspective belief is called self-perceptions of aging (SPA) and appears to have effects on psychological, behavioral, and physiological pathways (Levy, 2009). Previous studies have shown that more negative SPA was associated with adverse health outcomes (frailty, falls, hospitalizations, and decrease in activities of daily living; Moser et al., 2011; Sun et al., 2017), higher risk of death and shorter longevity (Sargent-Cox et al., 2014), higher depressive symptoms (Freeman et al., 2016). and worse cognitive functioning (Siebert et al., 2020). For older people who are in poor health, their attitude toward aging is likely to be more negative, which may put their health at even greater risk (Wurm et al., 2017). This study aimed to better understand how SPA is associated with frailty in a community-based sample of older adults to increase understanding of the underlying mechanisms that may exacerbate or delay the progression of frailty. Previous studies have shown that frailty is strongly associated with psychological, cognitive, and physical outcomes, such as depressive symptoms (Soysal et al., 2017), cognitive status (Searle and Rockwood, 2015), loneliness (Herrera-Badilla et al., 2015), and exercise (Kelaiditi et al., 2014). Depression is one of the most prevalent psychological distress in later life (Alexopoulos, 2005), and has been described as an antecedent of frailty (Chu et al., 2019). Furthermore, cognitive status and frailty were found to interact in an aging-related decline cycle (Robertson et al., 2013), the frailty among older adults is associated with the worse cognitive performance (Aguilar-Navarro et al., 2019). It is essential that higher levels of psychological health may help protect frailty and delay onset of frailty (Gale et al., 2014). Therefore, considering that depressive symptoms and cognitive status are strongly associated with SPA and frailty, depressive symptoms and cognitive status may also be the underlying psychological and cognitive pathways through which SPA affects frailty.

In the light of the above, the aim of this study is to conduct a cross-sectional study of older adults to determine the effect of self-perceptions of aging on frailty and internal relation mechanism using the SET as a framework. We performed this study to examine three hypotheses as follow: (a) SPA, loneliness, depressive symptoms, and locomotive function was positively associated with frailty. Cognitive status was negative associated with frailty. (b) SPA can influence frailty through depressive symptoms or cognitive status. To our knowledge, these possible mediators have not been studied in the Chinese context. Therefore, this study provides an opportunity to identify unique positive or negative aspects of Chinese older adults’ expectations and attitudes about their own aging, namely, SPA, to predict frailty, and explore the potential mechanisms between these two variables. Our research provides support for the consequences of aging stereotypes, providing support for Levy’s SET.

## Materials and Methods

### Participants and Procedure

From March to December 2019, a convenience sampling survey was conducted among people over 65 years old in the Xinxiang city community in Henan Province, China. All participants were older adults aged 65 years and above, taking part in the annual physical examination in the communities of the designated community health centers in Xinxiang, China. Individuals diagnosed with dementia by a doctor and participants who were unable to agree to participate in the study in person due to serious visual, auditory, or speech barriers were not included in this study.

Prior to the formal investigation, we introduced the research plan and specific procedures to the community health Service Center of Xinxiang City and obtained the approval of the institution. The investigators (8 postgraduate students in psychological and geriatric care) all underwent uniform standardized training prior to conducting the investigation. The investigators systematically described the purpose and process of the study to the participants to ensure that all participants knew the purpose of the survey and agreed to participate in the survey. During the formal investigation, investigators collected data through in-depth interviews. If a volunteer agreed to participate but had difficulty with reading or writing, the investigator assisted the respondent in filling out the form according to his/her wishes. Following testing, participants were thanked and compensated for their time. As compensation, each participant received a free breakfast.

### Sample Size

Depending on the requirements of the structural equation model (SEM), the sample size (N) and the amount of estimated limitations (parameters) need to be in the right proportion (McDonald and Ho, 2002). Kline believes the N:q ratio should be above 10 or even 20 (Kline, 1998). In this study, the N:q ratio rule was identified to be 20/1, q was identified to be 10. Therefore, a minimum sample size of 240 was calculated under the consideration of 20% invalid questionnaires. In this survey, we investigated 850 older adults. Due to lack of data, 28 participants were excluded. Thus, the final sample size was 822 (96.71%). The sample size met the requirements for SEM analysis.

### Measures

#### Socio-Demographic Variables

We assessed participants’ social demographic information (i.e., age, gender, educational level, marital status, and monthly income), health-related status (i.e., physical exercise, nutritional status, smoking history, and drinking history), and health conditions (i.e., anthropometric data and number of concurrent chronic diseases) through a demographic questionnaire devised by the researchers.

#### Self-Perceptions Assessment

SPA were assessed using the Chinese version of the Brief Aging Perceptions Questionnaire (B-APQ; Wang et al., 2021). The B-APQ is composed of 17 items that evaluate individuals’ understanding of aging along five different dimensions to determine whether a person’s attitudes are positive or negative (Sexton et al., 2014). Scores ranged between 17 and 85; the higher the overall score, the more negative an individual’s attitude toward aging. The Chinese version of the B-APQ was internally consistent and reliable (Cronbach’s α of 0.91; Wang et al., 2021).

#### Cognitive Status Assessment

Cognitive status was assessed using the Chinese version of the Mini-Mental State Examination (MMSE; Xiao-xuan et al., 2016), which consists of 30 items evaluating global cognition along the following dimensions: place and time orientation, attention and calculation, and memory recall. An MMSE score of 24 or lower usually indicates suspected cognitive impairment (Brown et al., 2020). The Chinese version of the MMSE was internally consistent and reliable (Cronbach’s α of 0.83; Xiao-xuan et al., 2016).

#### Loneliness Assessment

Loneliness was assessed using the Chinese version of the UCLA loneliness scale-8 (ULS-8; Zhou et al., 2012), an eight-item scale assessing an individual’s experience of loneliness. Total scores for this questionnaire range between 8 and 32. ULS-8 has shown satisfactory reliability and validity in evaluating loneliness among older Chinese adults, with a Cronbach α of 0.83 (Zhou et al., 2012).

#### Depressive Symptoms Assessment

Depressive symptoms were assessed using the Chinese version of the Patient Health Questionnaire-9 (PHQ-9; Wang et al., 2014). It was used to assess whether respondents had experienced sadness or depressive symptoms in the 2 weeks preceding the questionnaire survey. The sum of scores ranged from 0 to 27. A PHQ-9 score of ≥10 is considered to be the positive screening cutoff (Levis et al., 2019). PHQ-9 has been widely verified in different Chinese populations, with a Cronbach α of 0.86 in the general Chinese population (Wang et al., 2014).

#### Locomotive Function Assessment

The Japanese Orthopedic Association proposed the concept of “locomotive syndrome” for the prevention and treatment of locomotive organ diseases in 2007 (Nakamura, 2008). Locomotive syndrome is a condition in which damage to the locomotive organs (i.e., muscles, bones, and joints) leads to a decline in activities of daily living. Locomotive syndrome was assessed using the Chinese version of the Geriatric Locomotive Function Scale (GLFS-25; Ning et al., 2016). The sum of scores ranged from 0 to 100. A respondent with a GLFS-25 score of 16 or above is usually considered to be suffering from locomotive syndrome (Shinichi et al., 2014). The Chinese version of the GLFS-25 was internally consistent and reliable (Cronbach’s α of 0.92; Ning et al., 2016).

#### Frailty Assessment

Frailty was assessed using the Chinese version of the Tilburg Frailty Indicator (TFI; Dong et al., 2017), a self-rating scale developed based on the integral frailty model (Gobbens et al., 2010b). The scale measures three domains of frailty: physical frailty, psychological frailty, and social frailty. TFI is characterized by multi-dimensional structure, fast and easy to use, and accurate risk prediction of frailty adverse outcomes (Gilardi et al., 2018). Furthermore, TFI can better predict the overall functional status of older people’s bodies and provide evidence for clinical prevention and treatment of disease (Vrotsou et al., 2018). TFI is considered to be an appropriate instrument for assessing the frailty of older adults in the community (Sutton et al., 2016; Gilardi et al., 2018). TFI scores can range between 0 and 15, with the frailty cutoff being 5 (Gobbens et al., 2010a). TFI has been validated in populations from many countries all over the world, including Chinese older adults, with a Cronbach coefficient of 0.71 (Dong et al., 2017).

### Statistical Analysis

Our analytic approach involved two steps. First of all, descriptive statistics and univariate analysis were conducted with all variables by SPSS version 23.0. Spearman’s rank correlation analysis and multiple regression analysis were conducted to examine the relations between loneliness, depressive symptoms, self-perceptions of aging, locomotive function, cognitive status, and frailty. Then, the mediation model was tested using AMOS 23.0. A two-step procedure was used to analyze the mediation effect (Anderson and Gerbing, 1988). Firstly, the measurement model, which involved four latent variables, was tested to assess the goodness of fit represented by its explicit indicators. This study used several goodness-of-fit indices to evaluate model fit: Chi-square/degrees of freedom (*χ*^2^/df), root mean square error of approximation (RMSEA), goodness-of-fit index (GFI), comparative fit index (CFI), and the Tucker–Lewis index (TLI). A structural equation modeling (SEM) was considered acceptable when *χ*^2^/df was <5; RMSEA was <0.08; and GFI, CFI, and TLI were ≥ 0.90 (Schermelleh-Engel et al., 2003). Secondly, if the index of measurement model met the requirements, the maximum likelihood estimation examined the SEM. Besides the goodness-of-fit index, a bias-corrected percentile Bootstrap test was used to test the significance of the indirect effect, 5,000 repeated sampling with replacement was performed in the original data (*n* = 822), estimated 95% Confidence Interval (CI) for the indirect effect using a 2.5 percentile and a 97.5 percentile. When the bias-corrected 95% CI of bootstrap generated non-parametric estimation does not contain the number 0, the Point estimate is considered statistically significant (Hayes, 2013). When the value of *p* < 0.05, the result is considered to be statistically significant.

### Ethics Consideration

This study was conducted is in accordance with the Declaration of Helsinki. Before implementation of the research scheme, the study was approved by the local ethics committee (2019-HLPY-A001). Written informed consent was obtained from all participants in this study.

## Results

### Sample Characteristics

The participants’ socio-demographic data are presented in Table 1. The median age of the total sample was all around 70 years, and over half of the total sample was female (57.06%). The participants’ median body mass index (BMI) was M_BMI_ = 25 kg/m^2^. Normal BMI was reported in 305 (37.11%) cases. More than half of participants had obtained a high school education or above (52.80%). There were 396 (48.18%) cases of participants who were suffering from more than two chronic diseases simultaneously.

**Table 1:** Frailty among older adults with different sample characteristics (*N* = 822).

Characteristics	*Frequency (%)/Mean (SD)/[M(P75-P25)]*	TFI Univariate Analysis		
		*Mean rank*	*Z/χ^2^*	*P*
Age^a^, yr	70(7)			
Age group			34.280	<0.001^b^
60–69	390(47.45)	381.02		
70–79	362(44.03)	416.51		
≥80	70(8.52)	555.38		
Height (cm)	163.10(7.83)			
Weight (kg)	65(13)			
Waistline (cm)	84(10)			
BMI (kg/m^2^)	25(4)			
BMI group			5.999	0.112^b^
≤18.4	20(2.43)	521.63		
18.5–23.9	305(37.11)	413.00		
24.0–27.9	386(46.96)	412.10		
≥28.0	111(13.50)	385.47		
Gender			−4.463	<0.001^a^
Male	353(42.94)	370.15		
Female	469(57.06)	442.62		
Marital status			−5.033	<0.001^a^
Married	692(84.18)	393.97		
Divorced/Widowed/Unmarried	130(15.82)	504.83		
Educational level			32.451	<0.001^b^
Primary and below	154(18.73)	505.03		
Secondary	234(28.47)	403.45		
High and above	434(52.80)	382.65		
Monthly income (CNY)			21.048	<0.001^b^
<2,000	46(5.60)	502.48		
2,000–2,999	347(42.21)	416.06		
3,000–3,999	184(22.38)	443.05		
≥4,000	245(29.81)	364.26		
Physical exercise			−2.909	0.004^a^
Long-term adherence	722(87.83)	402.80		
Never or occasionally	100(12.17)	474.34		
Nutritional status			−3.529	<0.001^a^
Well-nourished	626(76.16)	395.63		
malnutrition	196(23.84)	462.19		
Smoking history			−2.026	0.043^a^
Yes	175(21.29)	380.19		
No	647(78.71)	419.97		
Drinking history			−1.954	0.051^a^
Yes	259(31.50)	388.34		
No	563(68.50)	422.15		
Number of concurrent chronic diseases			−4.196	<0.001^a^
≤1	426(51.82)	378.99		
≥2	396(48.18)	446.48		

*M*, Mean; SD, Standard deviation;

a range = 60–95 yr; BMI, Body Mass Index; CNY, Chinese Yuan.

a P derived from Mann-Whitney *U*-test;

b P derived from Kruskal–Wallis *H*-test.

Overall, the average B-APQ score was (40.95 ± 9.18). The median score of TFI, ULS-8, PHQ-9, GLFS-25, and MMSE were 3, 9, 1, 3, and 28, respectively. Among all participants, 21.53% reported frailty and 2.80% reported depressive symptoms. Cognitive impairment was found in 8.76% of respondents, and 8.27% showed signs of locomotive syndrome.

### Spearman’s Rank Correlation Analyses and Multiple Regression Analyses

We designed Table 2 to display the relationships of loneliness, depressive symptoms, self-perceptions of aging, locomotive function, cognitive status, and frailty. The results of Spearman’s rank correlation analyses suggested that all the variables were significantly correlated with one another. Frailty was significantly positively correlated with loneliness, depressive symptoms, self-perceptions of aging, and locomotive function (*r* = 0.267, *r* = 0.440, *r* = 0.481, *r* = 0.451; *p* < 0.001), and it also showed a significant negative correlation with cognitive status (*r* = −0.337, *p* < 0.001). SPA was significantly positively correlated with depressive symptoms (*r* = 0.327, *p* < 0.001), and it also showed a significant negative correlation with cognitive status (*r* = −0.175, *p* < 0.001).

**Table 2:** Spearman correlations for loneliness, depressive symptoms, self-perceptions of aging, locomotive function, cognitive status, and frailty.

Variable	*Mean (SD)/[M(P75-P25)]*	1	2	3	4	5	6
1. Frailty	3(2)	1					
2. Loneliness	9(3)	0.267^**^	1				
3. Depressive symptoms	1(3)	0.440^**^	0.269^**^	1			
4. Self-perceptions of aging	40.95(9.18)	0.481^**^	0.231^**^	0.327^**^	1		
5. Locomotive function	3(5)	0.451^**^	0.170^**^	0.426^**^	0.439^**^	1	
6. Cognitive status	28(3)	−0.337^**^	−0.057	−0.142^**^	−0.175^**^	−0.194^**^	1

** *p < 0.001*.

The statistically significant (*p* < 0.05) variables in the participants’ demographic variables and Spearman’s rank correlation analyses were entered into a multiple regression analysis for TFI using a stepwise selection procedure. The results showed that loneliness, depressive symptoms, self-perceptions of aging, locomotive function, and cognitive status were the five factors to be entered the regression equation, and the variance of joint explanation was 46.60%. Loneliness, depressive symptoms, self-perceptions of aging, locomotive function, and cognitive status were shown to explain frailty among older adults by 8.30, 31.0, 18.0, 25.30, and 17.30%, respectively. None of the participants’ social or demographic variables was an independent predictor of frailty in the multiple regression analysis (Table 3).

**Table 3:** Multiple regression results for frailty.

Variable	*Standardized β*	*SE*	*95% CI*
Loneliness	0.083	0.013	[0.014, 0.067]
Depressive symptoms	0.310	0.014	[0.124, 0.180]
Self-perceptions of aging	0.180	0.005	[0.019, 0.038]
Locomotive function	0.253	0.006	[0.039, 0.063]
Cognitive status	−0.173	0.015	[−0.120, −0.060]

*R*^2^ = 0.475, Adjusted *R*^2^ = 0.466; *F* = 52.183, *P* < 0.001.

### Structural Equation Modeling

We first constructed the relationship structure of all variables according to the results of the correlation matrix and multiple regression analyses. We further analyzed the influence of mediating latent variables on the relationship between independent variables and outcome variables, to determine whether they had weakened, strengthened, or no influence. The model of the relationships between self-perceptions of aging and frailty, as mediated by depressive symptoms and cognitive status, is shown in Supplementary Figure 1. The results show that the model has acceptable fit indices: *χ*^2^ = 303.398 (df = 85, *p* < 0.001), *χ*^2^/df = 3.569, GFI = 0.953, AGFI = 0.934, CFI = 0.939, TLI = 0.941, RMSEA = 0.056 (90% CI: 0.049, 0.063). Table 4 presents the bootstrap results, showing all the model’s standardized direct and indirect effects to be statistically significant.

**Table 4:** Bootstrapping total, direct, and indirect effects and 95% Confidence Interval (CI) for the mediation model.

Model pathways	Point estimate	Product of coefficients		Bias-corrected *95% CI*		Two-tailed	Ratio of total effect
(SPA Frailty)		*SE*	*Z*	Lower	Upper	Significance	
Total effect	0.845	0.060	14.083	0.735	0.969	<0.001	
Direct effect	0.306	0.079	3.873	0.148	0.456	<0.01	36.21%
Indirect effect	0.539	0.068	7.927	0.421	0.695	<0.001	63.79%

As illustrated in Supplementary Figure 1A, SPA (*β* = 0.306 and *p* < 0.001) and depressive symptoms (*β* = 0.714 and *p* < 0.001) had direct positive effects on frailty. Cognitive status (*β* = −0.483 and *p* < 0.001) had direct negative effects on frailty. The results from the bootstrap test for the significance of all pathways are shown in Table 4. Results for indirect pathways indicated that the indirect pathways between SPA and frailty through depressive symptoms were statistically significant (*β* = 0.391, 95% CI [0.027, 0.061], and *p* < 0.001). Furthermore, the indirect pathways between SPA and frailty through cognitive status were statistically significant (*β* = 0.148, 95% CI [0.009,0.024], and *p* < 0.001). Overall, the total effect of SPA on frailty was 0.539 through two indirect pathways (95% CI [0.421, 0.695] and *p* < 0.001). Based on these outcomes, it can be stated that depressive symptoms and cognitive status mediated the relationship between SPA and frailty and that this total mediating effect explains 63.67% of the total effect.

## Discussion

The burden of debilitating older people is expected to increase in low- and middle-income countries around the world due to rapidly aging populations (Hoogendijk et al., 2019). Therefore, it is imperative to clarify the potential mechanism of frailty in older adults, it is the main concern of healthcare policy and provision to formulate more effective intervention programs to prevent or slow down the development of frailty before it leads to substantial functional decline (Dent et al., 2019). The research purpose was to examine the effect of SPA on frailty and the mediating effect of depressive symptoms and cognitive status in this relationship.

Our study found that 177 older people in the community we sampled suffered from frailty (according to TFI score), and the prevalence of frailty was 21.53%. The results exceed the 18.0% prevalence of frailty previously detected by TFI among Chinese community-dwelling older people (Dong et al., 2017). This study shows that higher TFI scores were found among older women, those with low levels of education and low monthly incomes, those whose marital status is divorced or widowed or unmarried, those who have smoking history, those who never or only occasionally took part in physical exercise, those suffering from malnutrition, and those with two or more concurrent chronic diseases. This suggests that medical personnel should pay attention to the above characteristics in the population as they are possible frailty intervention targets.

According to SET, frailty was significantly positively correlated with loneliness, depressive symptoms, self-perceptions of aging, and locomotive function, and it also showed a significant negative correlation with cognitive status. Further multiple regression analysis showed that loneliness, depressive symptoms, self-perceptions of aging, locomotive syndrome, and cognitive status are key factors affecting the occurrence and development of frailty. This result further supports the previous research on frailty and its related factors (Kelaiditi et al., 2014; Herrera-Badilla et al., 2015; Searle and Rockwood, 2015; Carneiro et al., 2017). Therefore, health providers should pay more attention to these adjustable psychological and physical factors in order to delay the onset of frailty in older people.

By measuring individuals’ depressive symptoms and cognitive status levels, this study provides evidence of the internal mechanism of the influence of SPA on frailty. In this model of parallel multiple mediators for frailty, depressive symptoms and cognitive status played mediating roles, and weakened the effects of SPA. Our study found that older people with negative views of aging are more likely to suffer from frailty. This conclusion is consistent with that of Levy’s view on Aging Self-Stereotypes research (Levy, 2003). In addition, a longitudinal study also confirmed that people with a negative self-perceptions of aging were more likely to be frailer after 6 years (Warmoth et al., 2018). Therefore, this study suggests that perception of aging may be a contributing factor in the development of frailty and that promoting and maintaining positive perception of aging may provide a way to help individuals cope with health and frailty conditions.

The process of aging is a risk factor for frailty, cognition, and psychosocial function (Panza et al., 2018). Depressive symptoms are one of the most severe and prevalent mental disorders among older people (Gonçalves-Pereira et al., 2019). In this study, 2.80% of the community older adults sampled had depressive symptoms. We find that depressive symptoms play an important role in explaining the relationship between SPA and frailty. Psychological pathways suggest that more positive SPA is beneficial to relieve depressive symptoms, and thus delay the deterioration of frailty. On the contrary, when older adults have more negative attitudes toward aging, depressive symptoms have greater intrinsic psychological significance, which is a subjective unpleasant experience, which will trigger frailty worsening. As we introduced earlier, older people with passive SPA have an increased probability of future depressive symptoms (Freeman et al., 2016). In addition, consistent with previous findings, depressive symptoms conferred a higher risk for frailty worsening (Soysal et al., 2017), mainly due to their shared physiological etiology and the unhealthy lifestyle caused by depressive symptoms. Therefore, paying attention to and preventing depressive symptoms is of great significance for delaying frailty among older people.

Considering the various neurobiological mechanisms of aging and the changes in brain structure and function that occur with aging, human cognitive abilities will undoubtedly change with aging. In our study, the effect of SPA on frailty was also partly influenced by cognitive status. This result is supported by a previous study, which showed that the more positive a person’s attitude toward aging, the better their cognitive status, and the lower the risk of developing or forming frailty (Robertson and Kenny, 2016). We report that positive SPA may be a better predictor of cognitive health in older adults. This is consistent with the explanation in positive psychology including “how optimism and hope affect health” (Seligman and Csikszentmihalyi, 2000). In addition, Fredrickson’s broaden-and-build theory (Fredrickson, 1998) provides a mechanism by which positive beliefs and emotions may confer benefits to cognitive abilities. The correlation between frailty and cognitive impairment may be related to a common occurrence mechanism of both, such as genetic inheritance, chronic inflammation, malnutrition, mitochondrial dysfunction, oxidative stress, hypothalamic–pituitary–adrenal axis dysfunction, endocrine disorders, energy metabolism imbalance, and so on (Howrey et al., 2020). Therefore, good cognitive status can be considered a protective factor that can improve adverse effects in the pathway between self-perceptions of aging and frailty.

This study has some potential limitations that should be further interpreted. First, this study presents us with cross-sectional data, which does not allow us to determine causality. Second, we cannot track the health of patients, especially those with poor health outcomes (e.g., non-adaptive self-perceptions of aging, cognitive impairment, loneliness, depressive symptoms, and/or locomotive syndrome). These factors need further longitudinal study. Furthermore, physical frailty and mild cognitive impairment (MCI) often coexist, as these two syndromes share many common neuropathologies (Kwan et al., 2019). Therefore, future exploration of risk factors for cognitive frailty may lead to reversal of cognitive frailty outcomes. Despite these limitations, the current research helps us to understand the complex relationship between old people’s views on aging and frailty, giving it its value. The findings suggest that, in the context of China, depressive symptoms and cognitive status play a partial mediating role between SPA and frailty and that SPA, depressive symptoms, and cognitive status can be used as the targets of frailty intervention. This could provide a theoretical basis for improving frailty in future intervention studies. Future research must identify the predictive value of these determinants using longitudinal surveys in order to assess their usefulness within the context of intervention studies.

## Conclusion

This study first shows that the overall prevalence rate of frailty was 21.53%, and older people who view aging negatively tend to fall into a vicious spiral of frailty, depressive symptoms, and cognitive disorder. Degeneration is the result of the body’s natural development. It is worth noting that frailty is dynamic and reversible, and early screening and preventive intervention can delay or even reverse signs of frailty among older people. Our results suggest that community health service providers should implement timely evaluation of frailty status in older adults, help older adults revise their passive perspectives on aging, alleviate or prevent depressive symptoms, and improve cognitive status. This should be done to further delay the progress of frailty, improve clinical outcomes in older adults with frailty syndrome, and promote healthy aging.

## Data Availability Statement

The original contributions presented in the study are included in the article/ Supplementary Material, further inquiries can be directed to the corresponding author.

## Ethics Statement

The studies involving human participants were reviewed and approved by The First Affiliated Hospital of Xinxiang Medical University. The patients/participants provided their written informed consent to participate in this study. Written informed consent was obtained from the individual(s) for the publication of any potentially identifiable images or data included in this article.

## Author Contributions

YL, KY, JS, and HC participated in the concept and design of the study. KY and HH collected data and controlled quality. KY, YL, and JS drafted and edited the manuscript. KY, HH, and BZ performed the statistical analyses. All authors made substantial contributions to interpret data and revised the manuscript for important intellectual content. All authors contributed to the article and approved the submitted version.

## Funding

This research was supported by the Postgraduate Education Reform and Quality Improvement Project of Henan Province (HNYJS2020JD11).

## Conflict of Interest

The authors declare that the research was conducted in the absence of any commercial or financial relationships that could be construed as a potential conflict of interest.

## Publisher’s Note

All claims expressed in this article are solely those of the authors and do not necessarily represent those of their affiliated organizations, or those of the publisher, the editors and the reviewers. Any product that may be evaluated in this article, or claim that may be made by its manufacturer, is not guaranteed or endorsed by the publisher.
