# Supplementary material for: Depression and Cognition Mediate the Effect of Self-Perceptions of Aging Over Frailty Among Older Adults Living in the Community in China
Source: Front Psychol. 2022 Jun 16;13:830667. doi: 10.3389/fpsyg.2022.830667 (PMC9243527; doi:10.3389/fpsyg.2022.830667)
Supplement: Supplementary file 1 [file Image_1.pdf]

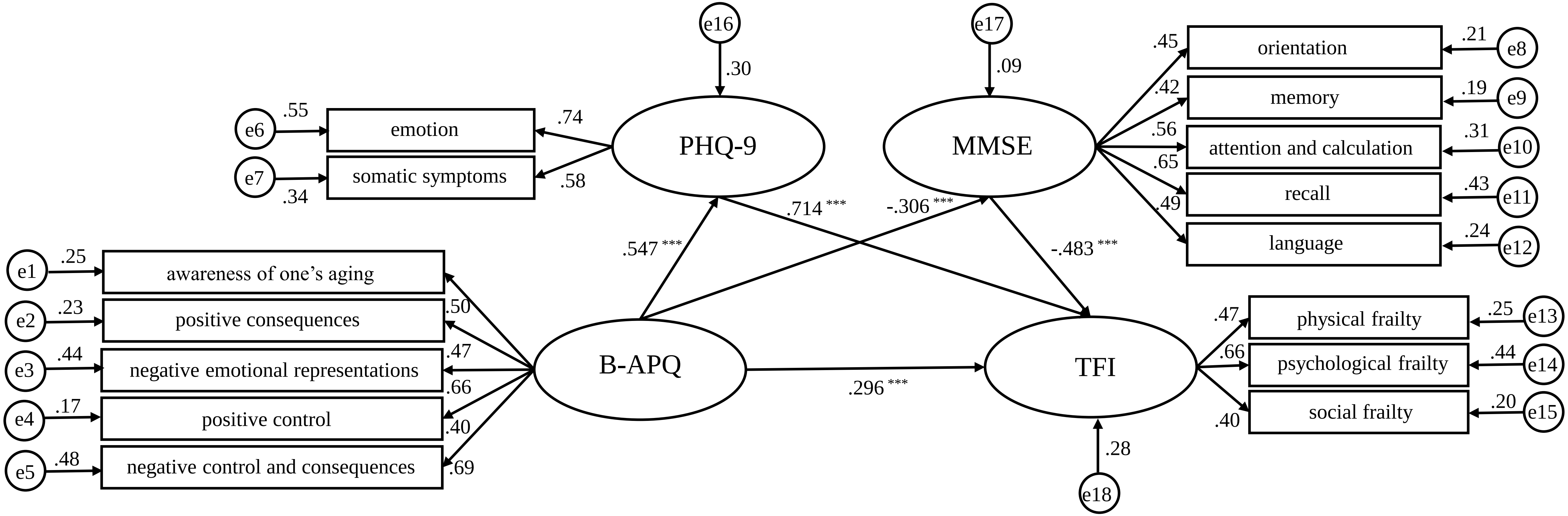

Supplementary image 1 The structural equation model on the relationship among self-perceptions of aging, depressive symptoms, cognitive status and frailty. Data are standardized regression coefficients. e1- e15, the measurement error of each observed variable to estimate latent variables; e16-e18, the residual that may affect the endogenous latent variables except the exogenous latent variables. *n*=822, ****P*<0.001.
